# Cultural Consensus Modeling to Understand South African Adolescent Girls' Attitudes, Awareness, and Uptake of Dual Protection Strategies

**DOI:** 10.1016/j.jadohealth.2020.07.003

**Published:** 2020-12

**Authors:** Jennifer L. Brown, Lochner Marais, Carla Sharp, Jan Cloete, Molefi Lenka, Kholisa Rani, Philile Marime, Irene Ditlhare, Refuwe Moqolo, Disebo Peterson, Jessica M. Sales

**Affiliations:** a Department of Psychiatry and Behavioral Neuroscience, University of Cincinnati College of Medicine, Cincinnati, Ohio; b Centre for Development Support, Economic and Management Sciences, University of the Free State, Bloemfontein, Republic of South Africa; c Department of Psychology, University of Houston, Houston, Texas; d Department of Behavioral Sciences and Health Education, Rollins School of Public Health, Emory University, Atlanta, Georgia

**Keywords:** Cultural consensus modeling, Dual protection, Pregnancy prevention, STI/HIV prevention, South African adolescents

## Abstract

**Purpose:**

This study used cultural consensus modeling to elucidate culturally relevant factors associated with dual protection use (strategies to prevent *both* pregnancy and sexually transmitted infection [STI]/HIV) among South African adolescent girls aged 14–17 years.

**Methods:**

In Phase 1, participants (N = 50) completed a free-listing survey assessing pregnancy and STI/HIV methods used by peers. In Phase 2, participants (N = 100) completed a rating survey to examine perceived peer acceptability of Phase 1 pregnancy and STI/HIV prevention methods. In Phase 3, qualitative individual interviews (N = 25) gathered in-depth information regarding the cultural acceptability of pregnancy and STI/HIV prevention strategies. In Phase 4, participants (N = 300) completed the Phase 2 rating survey for *individual* beliefs regarding the acceptability of pregnancy and STI/HIV prevention methods.

**Results:**

In Phase 1, 41 pregnancy and 29 STI/HIV prevention strategies, along with 16 factors influencing pregnancy prevention method acceptability were endorsed; male condoms were the most commonly endorsed pregnancy and STI/HIV prevention method. In Phase 2, using cultural consensus analysis, participants were consistent in the perceived acceptability of pregnancy and STI/HIV prevention methods (73.4% variance accounted for in single cultural model). In Phase 3, qualitative findings provided in-depth information regarding factors influencing commonly used pregnancy (e.g., injectable contraception) and STI/HIV (e.g., condoms) prevention methods. In Phase 4, a single cultural model was identified (56.3% variance accounted for), with similar acceptability ratings as Phase 2.

**Conclusions:**

A singular cultural model of pregnancy and STI/HIV prevention method acceptability was observed, with little awareness of dual protection. The findings highlight cultural factors for future culturally tailored dual protection interventions for South African adolescent girls.

**Implications and Contribution**

There was limited awareness of dual protection and greater acceptability of condoms as a pregnancy and sexually transmitted disease/HIV prevention method. The role of social and environmental factors was seen as influential for method acceptability. The results highlight factors that could be incorporated into culturally tailored dual protection interventions for South African adolescent girls.

See Related Editorial on p.737

South African adolescent girls experience elevated prevalence of both unintended pregnancies and sexually transmitted infections (STIs) including HIV, with significant health disparities experienced by black girls [[1], [2], [3]]. In South Africa, HIV rates increase rapidly throughout adolescence and young adulthood; for example, in a nationally representative sample, there was an HIV prevalence rate of 4.1% among individuals aged 15 years and 26.3% among individuals aged 24 years [4]. Recent epidemiological data indicate that 16% of South African adolescent girls between the ages of 15 and 19 years have begun childbearing [5]. National data point to high rates of sexual activity among 15- to 19-year-olds (83%), with only an approximate one-third using modern contraceptive methods [6]. Unintended teenage pregnancy and/or HIV result in numerous adverse health and socioeconomic consequences, including elevated risk of maternal and infant mortality, decreased educational attainment, exacerbation of poverty, and perpetuation of gendered power imbalances [7,8]. To reduce unintended pregnancies and STI/HIV and empower South African adolescent girls' reproductive health decision-making, interventions that consider relevant cultural factors are urgently needed for this population.

Reproductive health counseling to promote dual protection (use of one or more methods to prevent *both* pregnancy and STI/HIV) is included in South African national guidelines for reproductive health services [9]. However, the rates of reported dual protection use among South African women, and adolescents in particular, are low (e.g., 7% of adolescents using hormonal contraception reported co-use of condoms) [10]. In national data, injectable contraception (e.g., depot-medroxyprogesterone and norethisterone enanthate) has been identified as the most commonly used contraceptive method [6,11]. Despite emphasizing the provision of free male and female condoms and public health strategies to promote their use, the rates of consistent condom use among South African adolescents are also low [12,13].

There is limited empirical data characterizing factors associated with contraceptive practices, especially dual protection use, among South African adolescent girls [10,[14], [15], [16], [17]]. In one study, dual method use (hormonal contraception and condom use) was endorsed by a minority of adolescent girls (6.8%); correlates included discussing condom use with a sexual partner, number of lifetime sexual partners, and perceived difficulty accessing condoms [16]. The majority of research has focused on the correlates of male condom use, examining the role of condom use intentions [18,19], condom attitudes [15], and condom use self-efficacy [20]. Thus, the extant literature has focused on *individual-level* factors associated with contraceptive use, particularly condom use. Although studies of individual-level factors facilitate understanding of personal decision-making processes, there is a paucity of research examining the broader *cultural context* that may influence South African adolescent girls' contraceptive and dual protection practices [21].

Cultural consensus modeling (CCM) is an iterative methodology using an ethnographic approach to develop a culturally sensitive understanding of a given topic, with culture defined as learned and shared beliefs, behaviors, or norms held by a group [[22], [23], [24], [25]]. CCM produces a comprehensive understanding of a particular cultural domain [22,26] and has been used to describe cultural beliefs related to health outcomes (e.g., pubertal timing [26] and transactional sexual behaviors [23]). CCM first uses a free-listing approach to generate comprehensive lists of perceived behaviors or beliefs held by community members. In this way, CCM is locally appropriate with strengths of an ethnographic approach. However, ethnographic approaches are limited in their ability to ascertain whether identified beliefs or behaviors are shared across individuals or cluster across groups and whether identified cultural beliefs or behaviors predict outcomes of interest. The CCM approach addresses these limitations and uses a rigorous, multiphase mixed quantitative and qualitative approach to identify, from the ground up, a set of culturally relevant factors for a given outcome.

The aim of this study was to use CCM to elucidate culturally relevant factors associated with contraceptive practices, including dual protection use among South African adolescent girls by collecting qualitative and quantitative data across four iterative study phases.

## Methods

### Study design

**Figure 1:**
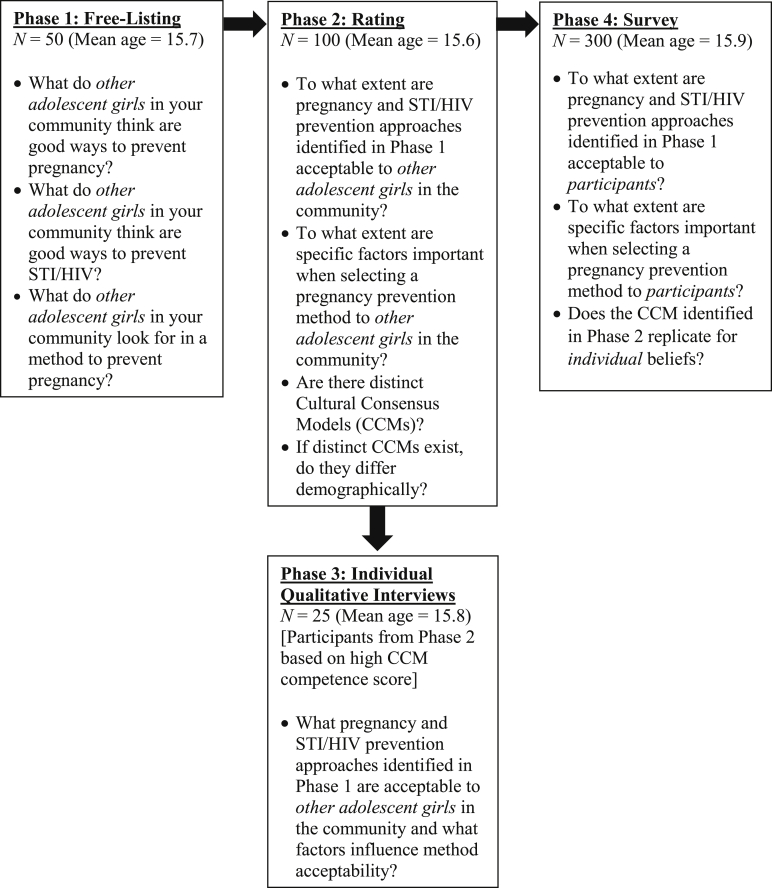
Study design and primary research questions for cultural consensus modeling methodology's four phases.

Figure 1 provides an overview of the study's iterative design and corresponding research questions across the four CCM study phases: (1) free-listing; (2) rating survey; (3) in-depth qualitative individual interviews; and (4) quantitative survey. All study protocols were approved by the University of the Free State's Institutional Review Board.

### Participants

South African adolescent girls were recruited from the urban areas of Mangaung Metropolitan Municipality in the Free State Province. Recruitment was conducted by Sesotho-speaking research staff through direct outreach efforts at community-based organizations and venues frequented by adolescents and through posting of fliers where interested individuals could contact research staff to learn more about the study. Eligibility for all study phases included being: (1) female; (2) between the ages of 14 and 17 years; (3) Sesotho-speaking; and (4) a resident in Mangaung. Written parental consent and written youth assent were obtained for all participants.

### Procedures

#### Phase 1

##### Free listing

Participants (N = 50) responded to demographic questions from the South African census [27] and to three free-listing prompts (Figure 1). Participants responded to two free-listing questions assessing methods used to (1) prevent pregnancy and (2) prevent STI/HIV and one examining factors that influence pregnancy prevention method selection. Data were collected via a tablet computer using REDCap's Mobile App [28].

#### Phase 2

##### Rating phase

Phase 2 asked a different sample of participants (N = 100) to rate the extent to which all methods identified in Phase 1 were valued. For each of the pregnancy and STI/HIV prevention methods identified in Phase 1, participants rated how acceptable they believed each method was among *other girls in their community* from very unacceptable (1) to very acceptable (5). For each of the factors influencing pregnancy prevention strategy selection identified in Phase 1, participants rated how important each factor was to *other girls in their community* from very unimportant (1) to very important (5). Data were collected via a tablet computer using REDCap's Mobile App [28].

#### Phase 3

##### In-depth qualitative interviews

Phase 3 conducted qualitative individual interviews with a subset of participants from Phase 2 (N = 25) who were highly consistent with the identified cultural consensus model based on competence scores. Study staff conducted interviews in Sesotho following a semistructured interview guide. Interview questions were designed to elucidate participants' perceptions of methods used to prevent pregnancy and STI/HIV by *other girls in their community*, the acceptability of identified methods, and factors associated with the use of a particular method. All interviews were digitally recorded, transcribed verbatim, and translated into English.

#### Phase 4

##### Quantitative survey

The survey was conducted with a convenience sample of young women (N = 300) who had not participated in previous phases. The questionnaire included the Phase 2 rating survey and corresponding response options, but unlike Phase 2, participants rated each item for their *individual beliefs* rather than perceived beliefs of other adolescents in the community. The primary outcomes of interest were participants' acceptability of pregnancy and STI/HIV prevention methods and factors influencing pregnancy prevention strategy selection. The survey also assessed the following domains: (1) demographics; (2) sexual behaviors; and (3) use of pregnancy and STI/HIV prevention methods. Data were collected via a tablet computer using REDCap's Mobile App [28].

### Data analytic approach

#### Phase 1

Free-listing responses for each of the three prompts (pregnancy prevention methods, factors influencing pregnancy prevention method selection, and STI/HIV prevention methods) were condensed to single master lists with frequencies generated.

#### Phase 2

The analytic approach examined the extent to which factors identified in Phase 1 were valued by participants and evaluated whether distinct cultural models (CMs) existed for different subpopulations. To identify CMs, cultural consensus analysis using UCINET software was conducted [29]. Cultural consensus analysis is similar to factor analysis but is conducted on respondents rather than items. Eigenvalues were generated to identify whether there was more than one CM present. Ratios between first and second eigenvalues exceeding 3:1 suggest the presence of a single CM [29,30]. Competence scores, the extent to which an individual participant was consonant with the identified CM, were calculated.

#### Phase 3

Qualitative analyses used a conventional content analysis approach using QSR International, NVivo 11 for Windows 2010 [31]. The analytic approach sought to (1) identify categories of specific pregnancy and STI/HIV prevention that were most acceptable; (2) examine factors associated with method acceptability; and (3) describe the frequency by which individual methods were endorsed. Transcripts were annotated with notes and labels for potential coding categories and subcategories related to pregnancy and STI/HIV prevention methods to develop the initial codebook. The codebook was used to code a randomly selected transcript with refinement of the initial coding classification scheme based on coding discrepancies and discussion of potential coding structure revisions using a standard iterative process [32,33]. Two independent raters then coded all transcripts using the finalized codebook to apply codes representing each theme or subtheme to text segments, with representative quotations identified. Coding discrepancies (i.e., differences in coded theme or subtheme for segmented text) were resolved by a third coder.

#### Phase 4

Descriptive statistics to characterize participants' lifetime sexual behavior engagement and contraceptive use were calculated. Analyses then examined the extent to which the CM identified in Phase 2 for *other adolescent girls* was replicated when examining participants' *individual* responses. As with Phase 2, consensus analysis on the ratings data using UCINET software was conducted [29]. Eigenvalues and the percent of variance accounted for were generated to identify whether one or more CMs were present. Exploratory bivariate analyses examined whether there were potential demographic and sexual history differences between respondents without and with negative competence scores (i.e., individuals not consonant with the CM).

## Results

### Phase 1

Frequencies of endorsed pregnancy and STI/HIV prevention methods and factors associated with the use of pregnancy prevention methods are shown in Table 1. Forty-one unique pregnancy prevention methods were identified, with the most frequently endorsed being condoms (78%), injectable contraception (58%), and contraceptive pills (22%). Sixteen factors were listed as important for selecting a pregnancy prevention method. The most frequently endorsed factors were availability of condoms (52%), availability of *free* condoms (52%), and availability of free injectable contraception (48%). Twenty-nine strategies to prevent STI/HIV were identified. The most frequently endorsed methods were male condoms (78%), abstinence (16%), and not touching blood without gloves (14%).

**Table 1:** Frequencies for Phase 1 free listing of pregnancy and STI/HIV prevention strategies (N = 50)^a^

	*n*	%
A. Pregnancy prevention methods		
Using condoms	39	78.0
Using injectable contraception	29	58.0
Using contraceptive pills	11	22.0
Abstaining from sex	8	16.0
Going to the public clinic for contraception	6	12.0
Not dating	6	12.0
Getting information from parents about pregnancy prevention	4	8.0
Being obedient to your parents	4	8.0
Having knowledge about consequences of pregnancy	4	8.0
Going to the doctor for contraception (paying to go to the doctor)	3	6.0
Prioritizing going to school and getting an education	3	6.0
Getting an implant	3	6.0
Learning how to refuse sex or say no to sex	3	6.0
Having sex with a person you trust	2	4.0
Abstaining from sex during menstruation	2	4.0
Resisting peer pressure	2	4.0
Going to the public clinic for information about how to prevent pregnancy	2	4.0
Taking the morning after-pill	2	4.0
Self-abortion	2	4.0
Taking advice from friends about sex	2	4.0
Ensuring others respect your body	2	4.0
Having an abortion	2	4.0
Avoiding alcohol	2	4.0
Receiving advice from elders	2	4.0
Getting an abortion, but not from a clinic (street abortion)	1	2.0
Receiving advice from older sisters or older girls	1	2.0
Using an IUD	1	2.0
Using laxatives for an abortion	1	2.0
Getting an abortion from a clinic	1	2.0
Using a traditional healer for an abortion	1	2.0
Going to the doctor for information about how to prevent pregnancy	1	2.0
Using withdrawal	1	2.0
Using herbs from traditional healers for contraception	1	2.0
Getting information from parents about values	1	2.0
Drinking water after sex	1	2.0
Avoiding drugs	1	2.0
Avoiding pornography	1	2.0
Getting information about pregnancy prevention from the media	1	2.0
Receiving sex education at school/from teachers	1	2.0
Having the belief that you do not want to get pregnant	1	2.0
Having well-behaved friends	1	2.0
Engaging in other activities such as sports	1	2.0
B. Importance of pregnancy prevention factors		
Availability of condoms in the community	26	52.0
Having free condoms available	26	52.0
Free injectable contraception	24	48.0
Availability of contraceptive pills in the community	13	26.0
Knowing about different contraception options	7	14.0
The ability to make your own decisions about pregnancy prevention	7	14.0
The ability to keep your abortion private (from parents or other people)	5	10.0
Information from older people	5	10.0
Having free clinic services	3	6.0
Not gaining weight	2	4.0
The type of pregnancy prevention method your friends are using	2	4.0
Having regular periods (regular menstrual cycles)	1	2.0
Balancing/weighing the advantages and disadvantages of a method	1	2.0
Minor side effects such as headache	1	2.0
No dangerous side effects like infertility	1	2.0
The ability to keep your contraceptive method private (from parents or other people)	1	2.0
C. STI/HIV prevention methods		
Using male condoms	39	78.0
Practicing abstinence	8	16.0
Not touching blood without gloves	7	14.0
Getting tested for STIs and HIV	6	12.0
Having a partner you know and trust	5	10.0
Having only one partner	5	10.0
Discussing your partner's HIV status	5	10.0
Knowing your partner's sexual history	4	8.0
Having HIV-infected individuals take antiretroviral medications	4	8.0
Having faithful relationships	4	8.0
Avoiding sex in exchange for gifts or money (blessers)	3	6.0
Not sharing needles	3	6.0
Not having sex with HIV-infected individuals	3	6.0
Using injectable contraception	3	6.0
Having the belief that sex is unacceptable	3	6.0
Avoiding sex with multiple partners	3	6.0
Not having sex with older partners	2	4.0
Getting more education and knowledge about STIs and HIV	2	4.0
Having HIV-infected individuals go to their doctors' appointments regularly	2	4.0
Using the female condom	2	4.0
Taking contraceptive pills	2	4.0
Avoiding peer pressure to have sex	2	4.0
Getting tested with your partner for STIs and HIV	2	4.0
Focusing on other activities	1	2.0
Checking the condom to make sure it is not expired or torn before using it	1	2.0
Focusing on your future	1	2.0
Taking pills after rape	1	2.0
Having HIV-infected individuals use condoms	1	2.0
Having the belief that sex is dangerous	1	2.0

IUD = intrauterine device; STI = sexually transmitted infection.

a Some minor wording modifications were made from the original English translation from Sesotho for ease of interpretation. For example, injectable contraception replaced the directly translated “pregnancy prevention needle.”

### Phase 2

Using the cultural consensus data analytic approach, a single CM was initially identified. There were two negative competence scores observed in the initial model, suggesting two respondents demonstrated poor fit with the model. The two participants with negative competence scores were removed, and the cultural consensus analysis was reperformed (N = 98). The resultant model indicated a single CM as the ratio of first-to-second eigenvalues exceeded the recommended ratio of 3:1 (largest eigenvalue = 31.04; second largest eigenvalue = 3.94) [29,30]. There were no negative loadings on the first factor suggesting good model fit. A majority of the variance (73.4%) was accounted for by the first factor. Thus, a single CM was supported by the data indicating a singular cultural consensus.

Participants with the highest consonance with the CM (based on competence scores) were invited to participate in Phase 3. Descriptive statistics are displayed in Table 2. Table 2 also includes the “culturally correct” answer key for each item; the Cultural Consensus Analysis score represents the CM's shared belief regarding the acceptability of a given pregnancy or STI/HIV prevention method by adjusting individual ratings based on one's competence with the CM. For example, *learning to refuse sex* as a pregnancy prevention method had a mean (standard deviation) of 4.5 (1.1), with mode = 5; the culturally correct rating was 4.71.

**Table 2:** Cultural consensus analysis (CCA) score and descriptive statistics for Phase 2 rating survey (N = 98^a^)

	CCA score	Mean (SD)	Mode
A. Pregnancy prevention methods			
Prioritizing going to school and getting an education	4.77	4.5 (1.2)	5
Being obedient to your parents	4.76	4.6 (.94)	5
Learning how to refuse sex or say no to sex	4.71	4.5 (1.1)	5
Having well-behaved friends	4.71	4.5 (1.1)	5
Receiving advice from elders	4.68	4.5 (1.1)	5
Ensuring others respect your body	4.66	4.5 (.97)	5
Having knowledge about consequences of pregnancy	4.57	4.3 (1.2)	5
Engaging in other activities such as sports	4.64	4.5 (1.2)	5
Getting information from parents about pregnancy prevention	4.54	4.3 (1.2)	5
Getting information from parents about values	4.48	4.2 (1.3)	5
Receiving sex education at school/from teachers	4.41	4.3 (1.2)	5
Going to the public clinic for information about how to prevent pregnancy	4.27	4.1 (1.3)	5
Receiving advice from older sisters or older girls	4.27	4.0 (1.3)	5
Using condoms	4.24	4.1 (1.3)	5
Going to the public clinic for contraception	4.24	4.1 (1.2)	5
Having the belief that you do not want to get pregnant	4.12	3.9 (1.5)	5
Going to the doctor for information about how to prevent pregnancy	3.94	3.7 (1.4)	5
Getting information about pregnancy prevention from the media	3.92	3.8 (1.3)	5
Avoiding alcohol	3.83	3.7 (1.4)	5
Not dating	3.82	3.7 (1.4)	5
Using injectable contraception	3.75	3.6 (1.4)	5
Avoiding drugs	3.74	3.6 (1.6)	5
Using contraceptive pills	3.58	3.5 (1.5)	5
Abstaining from sex	3.55	3.5 (1.5)	5
Going to the doctor for contraception (paying to go to the doctor)	3.45	3.4 (1.4)	5
Getting an Implant	3.39	3.4 (1.4)	5
Avoiding pornography	3.28	3.2 (1.6)	5
Resisting peer pressure	3.13	3.1 (1.6)	5
Abstaining from sex during menstruation	3.12	3.1 (1.7)	5
Taking the morning after-pill	3.12	3.1 (1.4)	3
Using an IUD	3.08	3.0 (1.4)	3
Drinking water after sex	3.06	3.1 (1.2)	3
Having sex with a person you trust	2.95	2.9 (1.4)	3
Getting an abortion from a clinic	2.63	2.6 (1.4)	1
Using withdrawal	2.62	2.6 (1.2)	3
Taking advice from friends about sex	2.51	2.6 (1.5)	1
Using herbs from traditional healers for contraception	2.17	2.4 (1.4)	1
Using a traditional healer for abortion	1.77	1.9 (1.1)	1
Using laxatives for an abortion	1.69	1.9 (1.2)	1
Getting an abortion, but not from a clinic (street abortion)	1.56	1.8 (1.1)	1
Self-abortion	1.55	1.7 (1.0)	1
B. Importance of pregnancy prevention factors			
Information from older people	4.73	4.6 (.87)	5
Having free condoms available	4.61	4.4 (1.1)	5
Knowing about different contraception options	4.54	4.4 (.97)	5
Having free clinic services	4.53	4.4 (1.1)	5
Availability of condoms in the community	4.43	4.2 (1.2)	5
Free injectable contraception	4.29	4.1 (1.2)	5
Availability of contraceptive pills in the community	3.89	3.8 (1.4)	5
Having regular periods (regular menstrual cycles)	3.52	3.4 (1.5)	5
Balancing/weighing the advantages and disadvantages of a method	3.46	3.5 (1.3)	4
No dangerous side effects like infertility	3.18	3.3 (1.5)	5
Minor side effects such as headache	3.14	3.3 (1.4)	5
The type of pregnancy prevention method your friends are using	3.05	3.0 (1.4)	3
Not gaining weight	2.94	2.8 (1.3)	3
The ability to make your own decisions about pregnancy prevention	2.71	2.8 (1.6)	1
The ability to keep your contraceptive method private (from parents or other people)	2.10	2.3 (1.5)	1
The ability to keep your abortion private (from parents or other people)	1.66	1.9 (1.4)	1
C. STI/HIV prevention methods			
Focusing on your future	4.74	4.6 (.98)	5
Getting more education and knowledge about STIs and HIV	4.73	4.5 (1.2)	5
Having HIV-infected individuals use condoms	4.69	4.5 (1.0)	5
Having HIV-infected individuals take antiretroviral medications	4.64	4.5 (.90)	5
Getting tested with your partner for STIs and HIV	4.61	4.4 (1.2)	5
Having only one partner	4.56	4.4 (1.1)	5
Having the belief that sex is dangerous	4.56	4.5 (1.1)	5
Getting tested for STIs and HIV	4.55	4.3 (1.3)	5
Having HIV-infected individuals go to their doctors' appointments regularly	4.53	4.3 (1.1)	5
Checking the condom to make sure it is not expired or torn before using it	4.49	4.4 (1.1)	5
Focusing on other activities	4.48	4.3 (1.1)	5
Using the female condom	4.39	4.3 (.99)	5
Using male condoms	4.29	4.2 (1.2)	5
Practicing abstinence	4.29	4.1 (1.4)	5
Not having sex with older partners	4.24	4.0 (1.6)	5
Having the belief that sex is unacceptable	4.21	4.1 (1.2)	5
Not having sex with HIV-infected individuals	4.14	3.9 (1.6)	5
Not touching blood without gloves	4.12	3.9 (1.6)	5
Having faithful relationships	4.08	3.8 (1.5)	5
Not sharing needles	4.05	3.8 (1.6)	5
Avoiding sex with multiple partners	3.98	3.8 (1.6)	5
Knowing your partner's sexual history	3.94	3.9 (1.2)	5
Having a partner you know and trust	3.89	3.8 (1.4)	5
Taking pills after rape	3.84	3.7 (1.5)	5
Taking contraceptive pills	3.69	3.6 (1.4)	5
Avoiding peer pressure to have sex	3.67	3.5 (1.6)	5
Using injectable contraception	3.58	3.5 (1.3)	4
Discussing your partner's HIV status	3.42	3.4 (1.5)	5
Avoiding sex in exchange for gifts or money (blessers)	1.87	1.9 (1.5)	1

IUD = intrauterine device; SD = standard deviation; STI = sexually transmitted infection.

a Descriptive statistics and Cultural Consensus Analysis (CCA) scores from final CCA model with N = 98.

As seen in Table 2, pregnancy prevention methods rated as highly acceptable within the CM were (1) being obedient to your parents; (2) learning how to refuse sex or say no to sex; and (3) having well-behaved friends. Highly rated factors for influencing pregnancy prevention methods include (1) information from older people; (2) having free condoms available; and (3) knowing about different contraception options. Highly acceptable STI/HIV prevention methods were (1) focusing on your future; (2) getting more education and knowledge about STIs and HIV; and (3) having HIV-infected individuals use condoms.

### Phase 3

Phase 3 used qualitative individual interviews with participants from Phase 2 who were highly consonant with the identified CM (N = 25; mean age = 15.8). Table 3 displays identified themes and exemplar quotations for pregnancy and STI/HIV prevention methods perceived to be most acceptable to *other adolescent girls* in the community by participants. For both pregnancy and STI/HIV prevention, condoms were the most commonly cited acceptable method, with acceptability influenced by high perceived efficacy (*“It is because according to research condoms provide 99% protection”).* A theme regarding condom use being the responsibility of male partners also emerged. Abstinence was commonly endorsed as both a pregnancy and STI/HIV prevention method, noting that refraining from sex entirely or until a later age was important. Injectable contraception methods and contraceptive pills were endorsed as highly acceptable methods to other adolescents, noting that examples of peers' use influenced acceptability. A minority of participants endorsed abortion, contraceptive implants, emergency contraception, and traditional medicine as pregnancy prevention approaches. Although not prevalent, male circumcision and STI/HIV testing were also identified as prevention strategies. Importantly, the theme of dual protection did *not* emerge from the qualitative interviews; instead, participants spoke about STI/HIV and pregnancy methods in isolation rather than their conjoint use.

**Table 3:** Phase 3 pregnancy and STI/HIV prevention method themes with frequencies and exemplar quotations (N = 25)

Pregnancy and STI/HIV prevention methods	*n*
A. Pregnancy prevention methods	
Abortion	3
• *“Others have an abortion, because they hear their boyfriends saying they will not support the child. Others have an abortion because their parents are heartless.”*	
Abstinence	8
• *“What they must do is abstain from sleeping with boys, because they have not reached that age yet.”*	
• *“I think a girl should just stay away from sex so that she is able to prevent pregnancy, STDs, and HIV.”*	
Condom	17
• *“It is because according to research condoms provide 99% protection…It means it is safe for 99%--meaning it is not totally safe, but it is safer than others.”*	
• *“For boys who ask for condoms I think they are taking responsibility for their own actions. They want to protect themselves all the time, they do not want to make mistakes.”*	
• *“From school as we know, we get taught about the fact that when we have sex with boys we must use condoms because they are available at clinics.”*	
Injectable contraception	19
• *“I think that is the only way, they use injections to prevent pregnancy.”*	
• *“I once heard about it from my friend. She told me about depo.”*	
• *“But then, I have a friend who always that is the one that she uses. I do not know it, but I think depo is for older people.”*	
Implant	3
• *“Yes, the girl who just left here went to the clinic and got an implant. But later on she got pregnant, and I asked her how come? Because you have an implant, she said it happened and she does not know how it happened. Then I decided I will not put in the implant because it is not certain.”*	
Emergency contraception (morning after pill)	5
• *“There are those pills that are called morning after pills. They are the right ones that you should take. Beside that then, you must not want use many things. You must avoid using injections. Always that the pills. If a boy ejaculates inside you, when you do not want to fall pregnant. Take morning after pills.”*	
Contraceptive pills	10
• *“They drink them [contraceptive pills] so that they do not fall pregnant when they go have sex.”*	
•*“Others would buy pills to prevent pregnancy…I do not know the names [of the pills].”*	
Traditional medicine/traditional healers	2
• *“Sometimes there are some girls who use traditional medicine. They sleep with their partners without using protection after that they drink the medication. The medicine is used to abort the baby.”*	
B. STI/HIV prevention methods	
Abstinence	7
• *“I think that you should abstain from having sex with boys. That's the only one.”*	
• *“It is to abstain from sex completely.”*	
Circumcision	2
• *“No but I think it is if the boy has gone for male circumcision at the hospital. After male circumcision he can have sex without using a condom because he will not infect his partner even if he is positive.”*	
Condom	18
• *“Because there is no other way you can use, other than condoms to prevent HIV and STIs.”*	
• *“My friends say it is to use a condom. Others say…there are others who say it is to use a condom, others say it is to use a female condom.”*	
• *“By using a condom and behaving well.”*	
STI/HIV testing	2
• *“They learn, I think that as girls to prevent STI and HIV we should not be sleeping with everyone, because you will never know how that person behaves. Or if you trust that person, the two of you should go together to a clinic, the two of you should get tested together so that you both know your status and are in a better position to manage it if anyone between the two of you is HIV positive.”*	

### Phase 4

The majority of participants did not endorse a lifetime history of vaginal sex (76.7%). Among those endorsing past vaginal sex (n = 56), the modal number of lifetime sexual partners was one; at last sex participants endorsed the use of condoms (78.6%), intrauterine device (IUD; 16.1%), implant (5.4%), injectable contraception (14.3%), patch (1.8%), and pills (10.7%), with 46.4% reporting dual condom and hormonal contraceptive method usage.

Using the cultural consensus data analytic approach, a single CM was identified as the ratio of first-to-second eigenvalues exceeded the recommended ratio of 3:1 (largest eigenvalue = 78.9; Second largest eigenvalue = 11.5) [29,30]. Descriptive statistics and the “culturally correct” answer key characterizing the acceptability of pregnancy and STI/HIV prevention methods and the importance of individual factors in selecting a method are displayed in Table 4. A majority of the variance (56.3%) were accounted for by the first factor or primary CM. However, there were 27 negative competence scores observed in the model, suggesting a subset of respondents (9%) demonstrated poor fit with the model. Exploratory analyses examined whether there were demographic or sexual history differences between participants with negative (n = 27) and positive (n = 273) competency scores. There were no differences between groups for age, number of individuals living in the household, current employment, access to private medical insurance, and lifetime history of vaginal sex. Among those with negative competency scores, there was a greater proportion not currently engaged in school (chi-square = 9.04; *p* = .02) and higher number of adults with a paid job in the household (*t* = −1.98; *p* = .05).

**Table 4:** Cultural consensus analysis (CCA) scores and descriptive statistics for Phase 4 survey (N = 300)

	CCA score	Mean (SD)	Mode
A. Pregnancy prevention methods			
Receiving advice from elders	4.84	4.3 (1.3)	5
Being obedient to your parents	4.83	4.3 (1.4)	5
Having well-behaved friends	4.76	4.2 (1.4)	5
Ensuring others respect your body	4.64	4.1 (1.4)	5
Prioritizing going to school and getting an education	4.56	4.0 (1.5)	5
Getting information from parents about values	4.52	3.9 (1.5)	5
Engaging in other activities such as sports	4.52	4.1 (1.3)	5
Having knowledge about consequences of pregnancy	4.50	4.0 (1.4)	5
Getting information from parents about pregnancy prevention	4.49	3.9 (1.4)	5
Learning how to refuse sex or say no to sex	4.48	3.9 (1.5)	5
Receiving sex education at school/from teachers	4.44	3.9 (1.5)	5
Using condoms	4.26	3.8 (1.6)	5
Having the belief that you do not want to get pregnant	4.25	3.9 (1.5)	5
Going to the public clinic for information about how to prevent pregnancy	4.16	3.7 (1.4)	5
Going to the public clinic for contraception	4.12	3.7 (1.5)	5
Receiving advice from older sisters or older girls	4.07	3.6 (1.6)	5
Avoiding drugs	3.96	3.6 (1.6)	5
Avoiding alcohol	3.90	3.6 (1.5)	5
Getting information about pregnancy prevention from the media	3.80	3.5 (1.4)	5
Not dating	3.60	3.3 (1.5)	5
Using injectable contraception	3.55	3.3 (1.5)	5
Having sex with a person you trust	3.53	3.3 (1.5)	5
Using an IUD	3.47	3.3 (1.5)	5
Resisting peer pressure	3.42	3.2 (1.5)	5
Taking the morning after-pill	3.30	3.2 (1.4)	3
Avoiding pornography	3.26	3.1 (1.7)	5
Using contraceptive pills	3.23	3.1 (1.6)	5
Drinking water after sex	3.18	3.1 (1.3)	3
Getting an Implant	3.12	3.0 (1.5)	3
Going to the doctor for information about how to prevent pregnancy	3.07	3.0 (1.6)	5
Going to the doctor for contraception (paying to go to the doctor)	3.04	2.9 (1.6)	5
Abstaining from sex	3.04	2.9 (1.5)	3
Abstaining from sex during menstruation	2.90	2.8 (1.7)	1
Getting an abortion from a clinic	2.51	2.6 (1.6)	1
Using herbs from traditional healers for contraception	1.92	2.2 (1.3)	1
Using withdrawal	2.47	2.6 (1.4)	1
Taking advice from friends about sex	2.13	2.4 (1.5)	1
Using laxatives for an abortion	1.52	1.9 (1.3)	1
Using a traditional healer for abortion	1.46	1.7 (1.2)	1
Self-abortion	1.42	1.8 (1.3)	1
Getting an abortion, but not from a clinic (street abortion)	1.36	1.8 (1.3)	1
B. Importance of pregnancy prevention factors			
Information from older people	4.76	4.4 (1.2)	5
Knowing about different contraception options	4.59	4.2 (1.2)	5
Having free condoms available	4.53	4.1 (1.4)	5
Having free clinic services	4.51	4.2 (1.3)	5
Availability of condoms in the community	4.49	4.1 (1.4)	5
Free injectable contraception	4.13	3.8 (1.4)	5
Availability of contraceptive pills in the community	3.76	3.5 (1.5)	5
Balancing/weighing the advantages and disadvantages of a method	3.62	3.4 (1.5)	5
Minor side effects such as headache	3.52	3.4 (1.4)	5
Having regular periods (regular menstrual cycles)	3.42	3.3 (1.4)	3
No dangerous side effects like infertility	3.25	3.2 (1.4)	3
The type of pregnancy prevention method your friends are using	2.88	2.9 (1.4)	3
Not gaining weight	2.64	2.7 (1.4)	3
The ability to make your own decisions about pregnancy prevention	2.46	2.5 (1.5)	1
The ability to keep your contraceptive method private (from parents or other people)	2.27	2.5 (1.5)	1
The ability to keep your abortion private (from parents or other people)	1.89	2.1 (1.4)	1
C. STI/HIV prevention methods			
Focusing on your future	4.82	4.3 (1.3)	5
Having only one partner	4.75	4.2 (1.3)	5
Having HIV-infected individuals use condoms	4.68	4.2 (1.3)	5
Getting more education and knowledge about STIs and HIV	4.65	4.2 (1.4)	5
Getting tested for STIs and HIV	4.65	4.2 (1.4)	5
Getting tested with your partner for STIs and HIV	4.57	4.1 (1.4)	5
Having HIV-infected individuals go to their doctors' appointments regularly	4.56	4.1 (1.3)	5
Checking the condom to make sure it is not expired or torn before using it	4.52	4.1 (1.4)	5
Focusing on other activities	4.51	4.1 (1.3)	5
Having HIV-infected individuals take antiretroviral medications	4.45	4.1 (1.4)	5
Having a partner you know and trust	4.45	4.1 (1.3)	5
Having the belief that sex is dangerous	4.34	3.9 (1.4)	5
Having faithful relationships	4.32	3.9 (1.4)	5
14. Using the female condom	4.28	3.9 (1.4)	5
Not touching blood without gloves	4.15	3.8 (1.7)	5
Knowing your partner's sexual history	4.22	3.9 (1.4)	5
Practicing abstinence	4.04	3.8 (1.3)	5
Using male condoms	3.92	3.7 (1.4)	5
Not having sex with older partners	3.90	3.5 (1.7)	5
Using injectable contraception	3.86	3.6 (1.4)	5
Taking contraceptive pills	3.81	3.5 (1.5)	5
Not sharing needles	3.80	3.6 (1.7)	5
Taking pills after rape	3.74	3.6 (1.4)	5
Having the belief that sex is unacceptable	3.68	3.5 (1.3)	3
Avoiding sex with multiple partners	3.71	3.4 (1.7)	5
Not having sex with HIV-infected individuals	3.67	3.4 (1.7)	5
Discussing your partner's HIV status	3.38	3.2 (1.5)	5
Avoiding peer pressure to have sex	3.37	3.2 (1.8)	5
Avoiding sex in exchange for gifts or money (blessers)	2.02	2.1 (1.6)	1

IUD = intrauterine device; STI = sexually transmitted infection.

As seen in Table 4, pregnancy prevention methods rated as highly acceptable within the CM were (1) receiving advice from elders; (2) being obedient to your parents; and (3) having well-behaved friends. Highly rated factors for influencing pregnancy prevention methods include (1) information from older people; (2) knowing about different contraception options; and (3) having free condoms available. Highly acceptable STI/HIV prevention methods were (1) focusing on your future; (2) having only one partner; and (3) having HIV-infected individuals use condoms.

## Discussion

Epidemiological data indicating elevated prevalence of unintended pregnancies and HIV among South African girls during adolescence through young adulthood highlight the importance of dual protection use to prevent both unintended pregnancies and STI/HIV [2,5]. CCM elucidated acceptable pregnancy and STI/HIV prevention methods and validated the identified single CM among Sesotho-speaking South African adolescent girls in the Free State Province that can be used to inform future intervention efforts. Although dual protection was not a theme in the CM, other specific pregnancy and STI/HIV methods (e.g., condoms and injectable contraception) were identified as highly acceptable across all study phases. Importantly, the role of adolescents' social network (e.g., affiliating with positive peers) and structural factors (e.g., access to free clinic services and condoms) emerged across the multiple phases of data collection as central to the acceptability of pregnancy and STI/HIV prevention methods.

To date, South Africa has emphasized HIV prevention messaging consistent with the Abstinence, Be Faithful, and Condom Use (ABC) approach, with less emphasis on dual protection to prevent both STI/HIV and unintended pregnancies. In line with a focus on HIV prevention messaging, ABC prevention themes emerged in the CCM as highly acceptable methods to prevent *both* pregnancy and STI/HIV, particularly the use of male condoms. Condoms represent the single method that afford both pregnancy and STI/HIV prevention. However, low rates of consistent condom use have been found across studies with South African adolescents [12,13] with differential availability of free condoms observed across provinces [34]. As noted by participants in the qualitative phase, condoms were also perceived to be the responsibility of boys and ultimately not within the control of adolescent girls. Although the complement of HIV prevention strategies has expanded beyond consistent condom use to include a cadre of biomedical approaches including pre-exposure prophylactic use of antiretroviral medications by HIV-uninfected individuals before HIV exposure, there was limited identification of such approaches. Instead, there was greater acceptability for HIV testing observed across study phases and some awareness of treatment as prevention and medical male circumcision as viable prevention approaches.

Injectable contraception was endorsed as highly acceptable by adolescents. This finding is consistent with previous national data pointing to injectable contraception as the most common method used (33%) relative to hormonal contraceptive pills (12%), male condoms (8%), IUDs (.8%), and tubal ligation (14%) [35]. Higher acceptability of injectable contraception and condoms may be due in part to the long-standing free availability of these methods in South African Department of Health facilities relative to more limited access to IUDs and implants [11]. Indeed, participants pointed to the importance of access to free clinic services and contraceptive methods as influencing adolescents' use. Policy initiatives to enhance access to the full complement of contraceptive options, including IUDs and implants, may further enhance the acceptability of these methods [36]. In addition to identifying specific contraceptive methods, participants also endorsed strategies not typically considered as pregnancy prevention methods (e.g., focusing on education). Such findings point to the important role of the social environment and norms around sexual behavior within this cultural context.

Unlike the extant literature that has focused largely on the examination of *individual-level* correlates of condom and contraceptive method use, the use of CCM resulted in a rich understanding of cultural factors associated with pregnancy and STI/HIV prevention methods that was consistent across all study phases. For example, adolescents emphasized the importance of focusing on one's future, affiliating with a positive peer group, and following the advice of parents or elders as influential to sexual decision-making. The Phase 2 CM accounted for a majority of the variance in ratings with few individuals having negative competence scores, suggesting strong model fit and a singular CM. Although the model for individual beliefs in Phase 4 indicated a single CM, there was less variance accounted for and a higher proportion of respondents who did not align with the model. Although there were few differences observed between participants with positive and negative competence scores, differences in current educational status and household composition may warrant further investigation. The results of this study also highlight the value of CCM for elucidating potential knowledge gaps regarding pregnancy and STI/HIV prevention methods (e.g., endorsement of contraceptive pills for STI/HIV prevention) to be addressed in future intervention efforts and the extent to which there is widespread penetration of public health policies and interventions (e.g., condom acceptability following sexual health education campaigns promoting their use). Furthermore, the results point to the importance of incorporating content to address social norms and structural factors to enhance the efficacy of pregnancy and STI/HIV prevention interventions [37].

Phase 4 data indicated that a minority of participants endorsed a history of sexual activity, suggesting this age period may be optimal for interventions to prevent unintended pregnancies and STI/HIV. Extant sexual health interventions for South African adolescents have focused primarily on HIV prevention [37,38]. With a primary HIV prevention emphasis, interventions have sought to increase male condom use, decrease the number of sexual partners, and address other HIV risk behaviors [37,38], with little emphasis placed on the use of efficacious pregnancy prevention strategies. The results highlight that condoms are perceived to be highly acceptable pregnancy and STI/HIV prevention methods. Consistent with trial data pointing to the importance of integrated STI/HIV and pregnancy prevention efforts (e.g., ECHO trial [39]), interventions highlighting *consistent* condom use coupled with the use of highly effective forms of contraception are needed. There is also a need to emphasize dual protection approaches using contraceptive methods and HIV prevention modalities that afford a greater deal of control by young women (e.g., pre-exposure prophylactic, implant, IUD, and injectable contraception). Given the importance of peers and trusted adults influencing sexual decision-making, interventions may wish to consider intervention approaches that may integrate the important role of one's social environment.

### Strengths and limitations

To our knowledge, this study represents the first to use CCM to examine the pregnancy and STI/HIV prevention method acceptability among an at-risk group of South African adolescent girls. CCM is a methodology that capitalizes on the strengths of an ethnographic approach coupled with rigorous quantitative and qualitative approaches to validate and expand on previous data collection phases. This approach facilitated a broader understanding of adolescent sexual health decision-making with respect to both pregnancy and STI/HIV prevention. However, because CCM is iterative in its approach, the acceptability of pregnancy and STI/HIV prevention methods were limited to those identified in the initial free listing phase. Furthermore, assessment of pregnancy and STI/HIV prevention methods individually during the initial free listing may have decreased the probability of reporting on dual protection approaches. Limitations include recruiting convenience samples of Sesotho-speaking adolescent girls in the Free State Province with parents who consented to study participation; thus, findings may not generalize to other South African adolescent girls. Only a minority of participants in Phase 4 were sexually active, so we are limited in our ability to assess the extent to which the acceptability of specific methods translates into uptake and use of methods or associated outcomes (e.g., incident pregnancy and HIV). Participants in Phase 4 may have been more likely to respond in a socially desirable manner because they were reporting on their own beliefs and behaviors rather than those of peers.

## Conclusions

Acceptability of dual protection strategies *did not* emerge as a prominent theme across all phases of data collection. Instead, themes consistent with ABC HIV prevention messaging emerged. Furthermore, participants endorsed the important role of social (e.g., affiliation with positive peers) and environmental factors (e.g., free clinic service access) as pregnancy and STI/HIV strategies. Young women placed less emphasis on the use of highly efficacious, modern contraceptive methods to prevent pregnancy. Given the paucity of evidence-based interventions to prevent both unintended pregnancies and STI/HIV for South African adolescents, the results of this study highlight the important cultural factors that could be incorporated into future culturally relevant dual protection interventions for South African adolescent girls.
